# Predictive Value of Neutrophil-to-Monocyte Ratio, Lymphocyte-to-Monocyte Ratio, C-Reactive Protein, Procalcitonin, and Tumor Necrosis Factor Alpha for Neurological Complications in Mechanically Ventilated Neonates Born after 35 Weeks of Gestation

**DOI:** 10.3390/pediatric16020027

**Published:** 2024-04-24

**Authors:** Daniela Mariana Cioboata, Marioara Boia, Aniko Maria Manea, Oana Cristina Costescu, Sergiu Costescu, Florina Marinela Doandes, Zoran Laurentiu Popa, Dorel Sandesc

**Affiliations:** 1 Department of Neonatology, “Victor Babes” University of Medicine and Pharmacy Timisoara, Eftimie Murgu Square 2, 300041 Timisoara, Romania; cioboata.daniela@umft.ro (D.M.C.); boia.marioara@umft.ro (M.B.); costescu.oana@umft.ro (O.C.C.); doandes.florina@umft.ro (F.M.D.); 2 Doctoral School Department, “Victor Babes” University of Medicine and Pharmacy Timisoara, Eftimie Murgu Square 2, 300041 Timisoara, Romania; 3 Department of Obstetrics and Gynecology, “Victor Babes” University of Medicine and Pharmacy Timisoara, Eftimie Murgu Square 2, 300041 Timisoara, Romania; costescu.sergiu@umft.ro (S.C.); popa.zoran@umft.ro (Z.L.P.); 4 Department of Obstetrics and Gynecology, Oravita City Hospital, 325600 Oravita, Romania; 5 Department of Anesthesia and Intensive Care, “Victor Babes” University of Medicine and Pharmacy Timisoara, Eftimie Murgu Square 2, 300041 Timisoara, Romania; sandesc.dorel@umft.ro; 6 Intensive Care Unit, “Pius Brinzeu” Emergency Clinical Hospital, 300041 Timisoara, Romania

**Keywords:** neutrophil-to-monocyte ratio, lymphocyte-to-monocyte ratio, inflammatory biomarkers, neurological complications, mechanical ventilation, neonates

## Abstract

This prospective study investigated the association between elevated neutrophil-to-monocyte ratio (NMR), lymphocyte-to-monocyte ratio (LMR), C-reactive protein (CRP), procalcitonin, and tumor necrosis factor-alpha (TNF-alpha) and the risk of developing neurological complications in mechanically ventilated neonates. The aim was to evaluate these biomarkers’ predictive value for neurological complications. Within a one-year period from January to December 2022, this research encompassed neonates born at ≥35 weeks of gestational age who required mechanical ventilation in the neonatal intensive care unit (NICU) from the first day of life. Biomarkers were measured within the first 24 h and at 72 h. Sensitivity, specificity, and area under the curve (AUC) values were calculated for each biomarker to establish the best cutoff values for predicting neurological complications. The final analysis included a total of 85 newborns, of which 26 developed neurological complications and 59 without such complications. Among the studied biomarkers, TNF-alpha at >12.8 pg/mL in the first 24 h demonstrated the highest predictive value for neurological complications, with a sensitivity of 82%, specificity of 69%, and the highest AUC (0.574, *p* = 0.005). At 72 h, TNF-alpha levels greater than 14.3 pg/mL showed further increased predictive accuracy (sensitivity of 87%, specificity of 72%, AUC of 0.593, *p* < 0.001). The NMR also emerged as a significant predictor, with a cutoff value of >5.3 yielding a sensitivity of 78% and specificity of 67% (AUC of 0.562, *p* = 0.029) at 24 h, and a cutoff of >6.1 showing a sensitivity of 76% and specificity of 68% (AUC of 0.567, *p* = 0.025) at 72 h. Conversely, CRP and procalcitonin showed limited predictive value at both time points. This study identifies TNF-alpha and NMR as robust early predictors of neurological complications in mechanically ventilated neonates, underscoring their potential utility in guiding early intervention strategies. These findings highlight the importance of incorporating specific biomarker monitoring in the clinical management of at-risk neonates to mitigate the incidence of neurological complications.

## 1. Introduction

Mechanical ventilation is a critical intervention in neonatology, providing life-saving respiratory support to neonates with inadequate pulmonary function [1,2]. This intervention, while indispensable, is associated with a range of potential complications due to the delicate nature of the neonatal respiratory system and the inherent risks of invasive support [3]. The advent of mechanical ventilation has revolutionized the care of critically ill neonates, enabling the survival of those with severe respiratory distress. However, the mechanical ventilation of neonates is not without its challenges and risks, particularly concerning the development of neurological complications [4,5].

Neurological complications in neonates are of significant concern, encompassing a spectrum of disorders such as hypoxic–ischemic encephalopathy, intraventricular hemorrhage, and periventricular leukomalacia [6,7]. These conditions are major contributors to morbidity and mortality in this population and can have profound impacts on long-term neurodevelopmental outcomes. The pathogenesis of these complications is multifactorial, involving direct mechanical effects, systemic inflammation, and oxygen toxicity, among other factors [8,9]. Thus, identifying neonates at high risk of neurological complications is a priority for clinicians, aiming to tailor interventions that might mitigate this risk.

In this context, the neutrophil-to-monocyte ratio (NMR) and lymphocyte-to-monocyte ratio (LMR), along with inflammatory biomarkers such as C-reactive protein (CRP), procalcitonin, and tumor necrosis factor alpha (TNF-α), emerge as potential tools for the early identification of at-risk neonates [10,11,12]. These markers offer insights into the neonate’s inflammatory status and immune response, which are intricately linked to the development of neurological complications [13,14]. Similarly, these markers proved to be valuable resources in studying other different conditions such as tumor pathology or inflammatory conditions [15,16].

The hypothesis is that elevated NMR, LMR, and inflammatory markers are associated with an increased risk of developing neurological complications in mechanically ventilated neonates. The objectives of this study are to assess the predictive value of these biomarkers for neurological complications and to establish a biomarker-based model that can guide clinical decision-making and intervention strategies. Through this research, we aim to contribute to the optimization of care for mechanically ventilated neonates, minimizing the risk of neurological complications and improving neurodevelopmental outcomes.

## 2. Materials and Methods

### 2.1. Study Design and Ethical Considerations

The current prospective observational cohort study was carried out over the span of one year, from January to December 2022, within the Neonatal Intensive Care Unit (NICU) at Louis Turcanu Children’s Emergency Clinical Hospital in Timisoara, focusing on neonates born at or beyond 35 weeks of gestational age, necessitating respiratory support through mechanical ventilation upon their admission on the first day of life.

This study was conducted in accordance with the ethical standards of the institutional research committee and with the 1964 Helsinki Declaration and its later amendments of ethical standards. The study protocol was reviewed by the Ethical Committee for Scientific Research, “Victor Babes” University of Medicine and Pharmacy of Timisoara, and approved on 28 September 2018, with the approval number 31. Informed parental consent was obtained from all individual parents of the participants included in this study.

The primary outcome of our study was the occurrence of neurological complications, including seizures, hypoxic–ischemic encephalopathy (HIE), and intracranial hemorrhage, in neonates receiving mechanical ventilation. Secondary outcomes focused on respiratory conditions and included the evaluation of persistent or severe respiratory illnesses requiring hospitalization, and the incidence of neonatal sepsis and other respiratory complications such as acute respiratory distress syndrome (ARDS), congenital pneumonia, pneumothorax, and pulmonary hemorrhage.

### 2.2. Inclusion and Exclusion Criteria

The inclusion criteria for this study comprised (1) late preterm and term newborns with a gestational age (GA) of 35 weeks or more, which includes late preterm infants defined as those born between 35 weeks of gestation and 36 weeks and 6 days of gestation (i.e., 239 to 259 days after the first day of the last menstrual period), term infants born at a GA of 37 weeks and 0 days of gestation through 41 weeks and 6 days of gestation, and post-term infants, categorized as those born after a 42-week GA; (2) newborns who required mechanical ventilation support upon admission, with no restrictions placed on birth weight; and (3) neonates who developed neurological complications such as seizures, HIE, and IVH.

Conversely, the exclusion criteria were distinctly defined to maintain this study’s focus and integrity: (1) the presence of severe congenital anomalies, particularly those affecting the cardiac, pulmonary, or central nervous systems, due to their potential independent impact on the neurological outcomes being studied; (2) neonates diagnosed with genetic syndromes, given the complex interplay between genetic factors and neonatal health outcomes; (3) infants who succumbed during the neonatal period or were lost to follow-up, as their outcomes could not be accurately assessed within this study’s parameters; (4) cases where informed consent for participation in this study and data collection was not obtained from the parents or legal guardians, adhering to ethical standards for human research.

### 2.3. Laboratory Investigations

For the purpose of conducting laboratory investigations, our study utilized a Sysmex XN-550 automated hematology analyzer, provided by Sysmex Corporation, Kobe, Japan, to perform a complete blood count (CBC). To facilitate this, 1 mL of peripheral venous blood was drawn from each participating neonate into a test tube containing EDTA (Ethylenediaminetetraacetic acid) as an anticoagulant. This method allowed for the precise quantification of the leukocyte formula, with measurements expressed in units of 10^3^/μL. The established normal range for various cell counts in neonates was as follows: WBCs ranged between 9 and 30 × 10^3^/μL, neutrophil counts spanned 1.5–22 × 10^3^/μL, lymphocytes were measured between 2 and 17 × 10^3^/μL, and monocytes fell within the range of 0.1–1.36 × 10^3^/μL.

Biochemical investigations to determine levels of CRP and procalcitonin were executed using either a Cobas Integra 400 Plus or Cobas e411 analyzer, both from Roche Diagnostics GmbH, Mannheim, Germany. For these tests, 2 mL of peripheral venous blood was collected in tubes free from anticoagulants but equipped with a separator gel. The defined normal range for CRP was established at 0–5 mg/L, and for PCT, at 0–0.5 ng/mL, which are critical markers for assessing the inflammatory status of the neonates under study.

TNF-alpha levels were ascertained by collecting 2 mL of peripheral venous blood in a vacutainer devoid of anticoagulants but containing separating gel. These samples were immediately centrifuged and subsequently stored at −70 degrees Celsius at the Hospital Analytical Laboratory until the time of processing. All TNF-alpha samples were analyzed in duplicate to ensure accuracy, utilizing an ELISA kit from DRG Instruments GmbH, Marburg, Germany, a method known for its precision in quantifying cytokine levels.

The collection of biological markers was strategically timed to capture critical postnatal intervals. The first time point (time point 1) was designated for blood tests collected within the initial hours following birth, while the second time point (time point 2) corresponded to blood tests conducted at 72 h of postnatal life.

Calculations for the NMR and the LMR were straightforward, fostering an efficient assessment of the neonates’ immune response. The LMR was determined by dividing the lymphocyte count by the monocyte count, whereas the NMR was calculated by dividing the neutrophil count by the monocyte count.

### 2.4. Respiratory Support and Mechanical Ventilation Description

The cohort of neonates included in this study required respiratory support that was managed through both invasive and non-invasive mechanical ventilation techniques, tailored to the severity of their respiratory distress and underlying medical conditions. In cases of invasive mechanical ventilation, the modes primarily utilized were Synchronized Intermittent Positive Pressure Ventilation (SIPPV) and Synchronized Mandatory Ventilation (SIMV). The ventilation parameters were set within a range to ensure adequate gas exchange and to reduce the work of breathing. Specifically, the Peak Inspiratory Pressure (PIP) was maintained between 18 and 25 cm H_2_O, and the Positive End-Expiratory Pressure (PEEP) was set between 5 and 8 cm H_2_O. The respiratory rate (RR) was adjusted to a range of 30–40 breaths per minute, with the Fraction of Inspired Oxygen (FiO_2_) tailored between 0.4 and 1.0, based on the neonate’s oxygenation status.

For neonates receiving non-invasive mechanical ventilation, nasal continuous positive airway pressure (NCPAP) was the initial modality of choice, with PEEP established at 5 cm H_2_O and FiO_2_ adjusted to maintain target oxygen saturation levels between 90 and 95%. When necessary, Intermittent Nasal Positive Pressure Ventilation (NIPPV) was employed, featuring PIP levels between 14 and 20 cmH_2_O, PEEP between 5 and 7 cm H_2_O, a respiratory rate of 10–40 breaths per minute, and an inspiration time ranging from 0.3 to 0.5 s.

The mechanical ventilators operational in our NICU were the Leoni Plus device (Löowenstein Medical SE & Co. KG, Bad Ems, Germany) and the Bellavista™ neo Ventilator (Vyaire Medical Inc., Chicago, IL, USA), ensuring high-quality respiratory support with a range of ventilation modes to cater to the specific needs of each neonate.

This study’s long-term outcome analysis leveraged data from the hospital’s database, encompassing records of subsequent admissions and consultations within the pediatric and pediatric neurology departments during the infants’ first 12 months. This follow-up period focused on identifying seizures occurring during this period, especially among neonates initially diagnosed with moderate-to-severe hypoxic–ischemic encephalopathy (HIE) and intracranial hemorrhage. The respiratory outcomes were also closely monitored, with particular attention on infants hospitalized more than twice for severe respiratory illnesses, indicating a possible chronic or recurrent condition.

Mechanical ventilation was initiated for various neonatal conditions, including neonatal sepsis, congenital pneumonia, severe neonatal asphyxia, meconium aspiration syndrome, respiratory distress syndrome (RDS), and transient tachypnea of the newborn (TTN). The immediate complications tracked in this study included moderate-to-severe hypoxic–ischemic encephalopathy, intracranial hemorrhage (including intraparenchymal and grade III-IV intraventricular hemorrhage), unilateral or bilateral pneumothorax, and pulmonary hemorrhage. For the long-term prognosis, up to approximately 12 months of age, this study focused on the onset of seizures requiring specific treatment and the occurrence of more than two episodes of severe respiratory illnesses necessitating hospitalization.

### 2.5. Statistical Analysis

The data management and analysis were conducted utilizing the statistical software SPSS version 26.0 (SPSS Inc., Chicago, IL, USA). Sample size calculation and study power were determined based on a convenience sampling method. Aiming for a power of 80% to detect this difference at a 5% significance level and 10% margin of error, the required sample size was calculated at a minimum of 62 patients. Normality distribution was tested with the Kolmogorov–Smirnov test. Continuous variables that were normally distributed were represented as the mean ± standard deviation (SD), while categorical variables were expressed in terms of frequencies and percentages. The Student’s *t*-test compared two means between the normally distributed data. The Chi-square test was utilized for the categorical variables. The best cutoff value, sensitivity, specificity, area under the curve (AUC), and the Receiver Operating Characteristic (ROC) were calculated to determine the prediction value of the proposed parameters. The prospective collection of data allowed for the calculation of sensitivity, specificity, and predictive values with the intent to establish biomarkers that could potentially predict neurological complications. A *p*-value threshold of less than 0.05 was set for statistical significance. All results were double-checked to ensure accuracy and reliability.

## 3. Results

### Patient Demographics

The final analysis included 26 neonates with neurological complications and 59 without, revealing that gender distribution between neonates with neurological complications (69.23% male, 30.77% female) and those without (67.80% male, 32.20% female) showed no significant difference (*p* = 0.895). None of the 85 cases succumbed or were lost to follow-up in the neonatal period. Similarly, no significant differences were found in gestational age (36.69 ± 1.59 weeks for those with complications vs. 36.29 ± 1.82 weeks for those without; *p* = 0.335).

Though not statistically significant, neonates with neurological complications had a higher mean birth weight (2941 g) compared to those without complications (2626 g), with a *p*-value of 0.073. The statistical analysis showed a significant difference in APGAR (appearance, pulse, grimace, activity, and respiration) scores at both 1 min (5 for those with complications vs. 7 for those without; *p* < 0.001) and 5 min (6 vs. 7; *p* < 0.001).

No significant differences were observed in the rates of monitored pregnancy (57.69% in those with complications vs. 61.02% in those without; *p* = 0.773), cesarean birth (65.38% vs. 69.49%; *p* = 0.707), meconium aspiration (11.54% vs. 8.47%; *p* = 0.655), and transient tachypnea (30.77% vs. 38.89%; *p* = 0.468) between the two groups, as presented in Table 1.

The analysis demonstrated no significant difference in the incidence of neonatal sepsis between the groups (19.23% in those with neurological complications vs. 18.64% in those without; *p* = 0.949). Similarly, no significant differences were found in the rates of acute respiratory distress syndrome, congenital pneumonia, pneumothorax, pulmonary hemorrhage, and recurrent pneumonia between neonates with and without neurological complications, respectively.

However, a significant association was observed with neonatal asphyxia, where a markedly higher percentage of neonates with neurological complications experienced asphyxia (38.46%) compared to those without complications (8.47%), with a *p*-value of <0.001. Regarding the interventions, the use of non-invasive positive pressure ventilation (nIPPV) was significantly higher in neonates with neurological complications (50.00%) compared to those without (25.42%), with a *p*-value of 0.026. However, the use of nasal continuous positive airway pressure (nCPAP) or nIPPV and synchronized intermittent mechanical ventilation (SIMV) or invasive positive pressure ventilation (IPPV) did not show significant differences between the groups (*p* = 0.246 and *p* = 0.827, respectively). The duration of ventilation exceeding 7 days was also not significantly different, although there was a trend towards a longer duration in neonates with neurological complications (57.69% vs. 38.89%; *p* = 0.109), as seen in Table 2.

The analysis of C-reactive protein in the first 24 h levels showed higher averages in neonates with neurological complications (11.39 mg/L) compared to those without (8.29 mg/L), although this difference was not statistically significant (*p* = 0.245). Similarly, procalcitonin levels were elevated in the group with neurological complications (16.16 ng/mL) versus the group without (9.44 ± 15.64 ng/mL), with a *p*-value of 0.059.

White blood cell counts were significantly higher in neonates with neurological complications (20.70 × 10^3^/μL) compared to those without complications (15.39 × 10^3^/μL), with a *p*-value of 0.032. A significant difference was also observed in the lymphocyte count, which was higher in neonates with neurological complications (4.61 × 10^3^/μL) compared to those without (2.41 × 10^3^/μL), with a *p*-value of 0.004.

Neutrophil and monocyte counts, although different between the two groups, did not reach statistical significance (*p* = 0.131 for neutrophils and *p* = 0.868 for monocytes). Significantly, this study found that both the neutrophil-to-monocyte ratio and the lymphocyte-to-monocyte ratio were higher in neonates with neurological complications (NMR: 7.59, LMR: 6.18) compared to those without (NMR: 5.44, LMR: 2.63), with *p*-values of 0.035 and 0.027, respectively. Tumor necrosis factor-alpha (TNF-alpha) levels were also significantly higher in those with neurological complications (15.76 pg/mL) compared to those without (11.06 pg/mL), with a *p*-value of 0.013 (Table 3).

The laboratory analysis at 72 h revealed a statistically significant higher mean CRP level in neonates with neurological complications (12.64 mg/L) compared to those without (6.89 mg/L), with a *p*-value of 0.036. Similarly, procalcitonin levels were significantly higher in the group with neurological complications (8.56 ng/mL) as opposed to those without (4.43 ng/mL), with a *p*-value of 0.042. White blood cell counts were also found to be significantly higher in neonates with neurological complications (17.35 × 10^3^/μL) compared to those without (13.51 × 10^3^/μL), with a *p*-value of 0.020, indicating an association between elevated WBCs and neurological complications.

Although the lymphocyte count was higher in neonates with neurological complications (4.59 × 10^3^/μL) versus those without (3.66 × 10^3^/μL), this difference was not statistically significant (*p* = 0.181). A significant finding was the mean neutrophil count, which was markedly higher in neonates with neurological complications (7.58 × 10^3^/μL) compared to those without (3.41 × 10^3^/μL), with a *p*-value of <0.001.

No significant difference was found in monocyte counts between the two groups (*p* = 0.145). The NMR and the LMR were also significantly different, with NMR being higher in neonates with neurological complications (5.42) compared to those without (3.83), *p* = 0.049, and LMR significantly higher in the complications group (4.22) versus the no-complications group (2.71), *p* = 0.005. Lastly, TNF-alpha levels were significantly higher in neonates with neurological complications (19.59 pg/mL) compared to those without (13.65 pg/mL), with a *p*-value of 0.022 (Table 4).

For the 24 h measurements, the analysis demonstrated that the best cutoff value for CRP was >10.7 mg/L, with a sensitivity of 68% and specificity of 62%, though the area under the curve (AUC) of 0.431 and a *p*-value of 0.168 suggest that this marker was not a strong predictor at this timeframe. Similarly, procalcitonin with a cutoff of >12.2 ng/mL showed a sensitivity of 66% and specificity of 59%, with an AUC of 0.429 and a *p*-value of 0.183, indicating limited predictive value.

In contrast, the NMR at a cutoff of >5.3 demonstrated a higher sensitivity of 78% and specificity of 67%, with a more favorable AUC of 0.562 and a significant *p*-value of 0.029, suggesting its usefulness as a predictive marker. The LMR with a cutoff of >4.2 also showed promising predictive capabilities with a sensitivity of 71%, specificity of 64%, an AUC of 0.558, and a *p*-value of 0.038. Tumor necrosis factor-alpha (TNF-alpha) emerged as a strong predictor with a cutoff of >12.8 pg/mL, achieving a sensitivity of 82%, specificity of 69%, the highest AUC of 0.574 among the 24 h measurements, and a significant *p*-value of 0.005.

**Figure 1:**
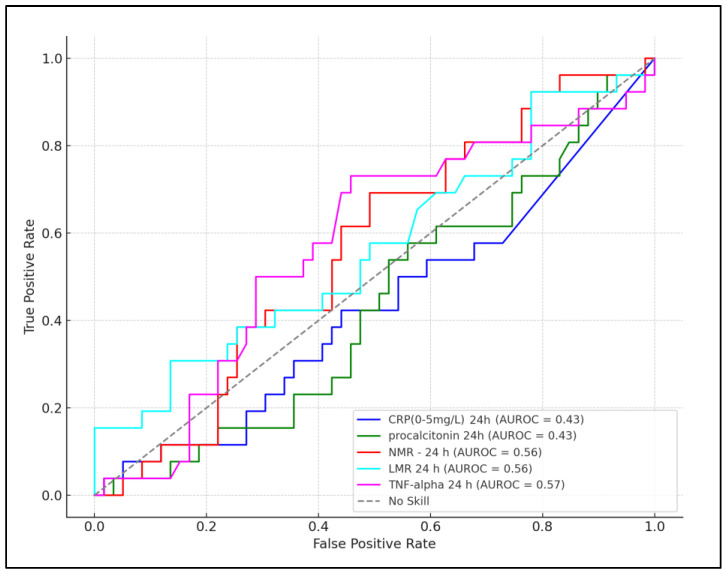
ROC plot for prediction role of laboratory parameters measured in the first 24 h, for neurological complications.

**Figure 2:**
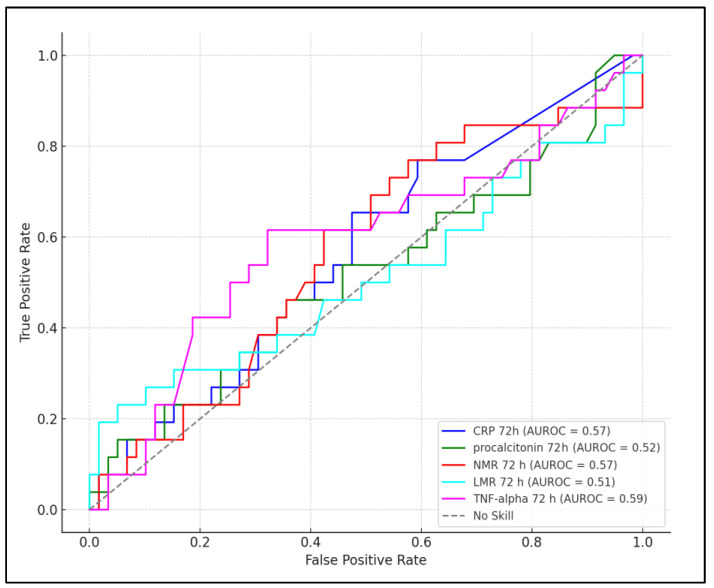
ROC plot for prediction role of laboratory parameters measured at 72 h, for neurological complications.

At 72 h, the predictive value of the biomarkers appeared to improve for certain parameters. CRP with a cutoff of >15.4 mg/L showed increased sensitivity and specificity (74% and 66%, respectively) and a higher AUC of 0.570 with a significant *p*-value of 0.032. Procalcitonin at a cutoff of >0.32 ng/mL offered a sensitivity of 70% and specificity of 61%, with an AUC of 0.520 and a *p*-value of 0.059. NMR remained a reliable predictor with a cutoff of >6.1, showing a sensitivity of 76%, specificity of 68%, an AUC of 0.567, and a significant *p*-value of 0.025. The LMR, at a cutoff of >3.7, had a sensitivity of 68%, specificity of 63%, and an AUC of 0.510 with a *p*-value of 0.076, indicating a slight decrease in its predictive performance compared to the 24 h timeframe. TNF-alpha maintained its strong predictive value with a higher cutoff of >14.3 pg/mL, achieving the highest sensitivity (87%) and specificity (72%) at 72 h, along with the highest AUC of 0.593 and a highly significant *p*-value of <0.001, as presented in Table 5, and Figure 1 and Figure 2.

C-reactive protein above its best cutoff value was associated with a hazard ratio (HR) of 1.41, indicating a 41% increase in the risk of developing neurological complications compared to neonates with CRP levels below the cutoff (*p*-value = 0.030). Procalcitonin levels above the best cutoff demonstrated a hazard ratio of 1.30, suggesting a 30% increased risk for neurological complications. However, the association was not statistically significant. The neutrophil-to-monocyte ratio above its best cutoff had a significant hazard ratio of 2.16, meaning that neonates with NMR values above the cutoff were more than twice as likely to develop neurological complications. This significant association, with a 95% CI of 1.18 to 4.09 and a *p*-value of 0.022, highlights NMR as a strong predictive marker.

The lymphocyte-to-monocyte ratio (LMR) also showed a significant hazard ratio of 1.94, indicating nearly double the risk of developing neurological complications for neonates with LMR values above the cutoff (*p*-value = 0.008). Tumor necrosis factor-alpha (TNF-alpha) demonstrated the most significant hazard ratio of 3.32, indicating that neonates with TNF-alpha levels above the best cutoff were over three times more likely to develop neurological complications. This association was highly significant, with a 95% CI of 2.06 to 6.39 and a *p*-value of <0.001, as presented in Table 6.

## 4. Discussion

This study’s findings, particularly those related to the inflammatory markers and cell count ratios, illuminate the complex interplay of neonatal physiological responses to stress and infection, and their potential predictive value for neurological complications in mechanically ventilated neonates. The initial 24 h measurements, while not uniformly statistically significant across all parameters, indicated trends toward higher levels of CRP and procalcitonin in neonates with neurological complications. These trends, although not reaching conventional levels of statistical significance, hint at an underlying inflammatory response that could be more pronounced in neonates at risk for or experiencing neurological complications, as indicated by other studies, although on adult patients [17,18].

By the 72 h mark, the statistical significance of CRP and procalcitonin levels was established, alongside a marked increase in white blood cell and neutrophil counts, underscoring a more pronounced inflammatory response. This evolution over time not only strengthens the argument for these markers as potential indicators of neurological complications but also suggests that the window for predictive intervention may extend beyond the first hours of life. The significant findings related to NMR and LMR offer a compelling case for the inclusion of these ratios in routine neonatal screenings, potentially providing clinicians with a more nuanced understanding of a neonate’s risk profile for neurological complications. Similarly, other recent studies identified NMR and LMR as potential predictors, although for different complications, such as threatened abortion and neonatal sepsis [19,20].

Regarding TNF-alpha, the most accurate predictor for neurological complications in the early neonatal period, as identified by our study, one meta-analysis highlighted a moderate diagnostic accuracy of TNF-α in identifying both early-onset and late-onset neonatal complications and sepsis. Findings indicated a sensitivity of 0.66 and specificity of 0.76 for early-onset sepsis, while late-onset sepsis showed a sensitivity of 0.68 and specificity of 0.89. Moreover, a notable higher sensitivity (0.84) and specificity (0.83) were observed in the northern hemisphere group [21]. Moreover, Kim et al.’s study on neural progenitor cell (NPC) therapy for hypoxic–ischemic brain injury revealed that pretreating NPCs with TNF-α significantly enhanced their survival and therapeutic efficacy [22]. TNF-α pretreatment increases the expression of protective proteins and neurotrophic factors, leading to improved neuroprotection and reduced brain damage. This approach presents a promising strategy to enhance the benefits of NPC therapy in treating brain injuries by improving the survival and function of transplanted cells.

Other significant predictors such as NLR and LMR were also identified by other researchers as important predictors for neonatal sepsis. In a study by Li et al. involving 1480 neonates, it was found that those with sepsis had a significantly elevated neutrophil–lymphocyte ratio, with sepsis likelihood increasing from 41.6% at an NLR below 0.91 to 66.2% above 1.88. An NLR of 1.62 was identified as the optimal threshold for sepsis prediction, presenting an area under the curve of 0.63, underscoring NLR as an independent risk factor for neonatal sepsis [23].

Nevertheless, all these markers should be considered in association with mechanical ventilation, as the focus of the current study aimed. Thus, a study by Bohrer et al. [24] evaluated the impact of mechanical ventilation on the levels of various cytokines in term and late preterm neonates without previous mechanical ventilation or ventilatory support history. The study found that among 19 newborns studied, with an average gestational age of 35.8 weeks and birth weight of 2280 g, significant changes were observed: pro-inflammatory cytokines like IL-8 increased 2.5-fold, IL-1beta by 7.5-fold, and TNF-alpha by 10-fold, while the anti-inflammatory cytokine IL-10 decreased by 90%. IL-6 levels remained consistent, though it increased in 89.4% of infants.

Regarding the long-term implications of neurological complications, a study by Numis et al. [25] found that neonatal seizures linked to brain injury were associated with an increased risk of developing epilepsy in childhood. Analyzing a cohort of 26 newborns at risk for hypoxic–ischemic encephalopathy, the study noted diffuse alterations in cytokine levels, with significant associations between higher levels of pro-inflammatory cytokines, specifically IL-6 and TNF-α within the IL-1β pathway, and the later onset of epilepsy. Out of the 17 patients followed for more than two years, 24% developed epilepsy, suggesting that elevations in certain pro-inflammatory cytokines could serve as indicators for the potential development of epilepsy in patients with neonatal encephalopathy.

Studies by Wu et al. [26] and Mehta et al. [27] highlight the significant role of hematological ratios like the NMR and LMR in understanding the immune response related to neurological complications in adults, potentially stemming from neonatal neurological conditions. Wu et al. found that these ratios, particularly LMR, were significantly correlated with plaque enhancement in intracranial atherosclerotic stenosis, indicating their utility in predicting vascular instability that could lead to cerebral ischemic events. Similarly, Mehta et al. reported that NMR and LMR are indicative of neuroinflammation associated with Alzheimer’s disease pathology, with changes in these ratios reflecting an imbalance in innate versus adaptive immunity, which correlates with β-amyloid deposition and cognitive decline. These findings suggest that early life alterations in immune response markers could have long-standing effects, potentially leading to or exacerbating adult neurological conditions, thus underscoring the importance of monitoring these ratios as part of a comprehensive approach to long-term neurological health.

Critical to the discussions around these findings is the consideration of the biological plausibility underlying these associations. The mechanisms through which elevated inflammatory markers and cell count ratios increase the risk of neurological complications in neonates may be rooted in the inflammatory cascade’s impact on brain development and function. Therefore, our study offers valuable insights that may influence clinical practice. The biomarkers studied have the potential to be used in real-time to guide clinical decisions regarding the monitoring and management of neonates at risk of developing neurological complications. Future research should aim to elucidate these mechanisms further, validate these findings in larger cohorts, and explore the potential for these markers to inform clinical practice, with the ultimate goal of improving outcomes for mechanically ventilated neonates.

We selected the 24 h and 72 h time points for sample collection based on the typical onset times of neurological complications and aligning with other studies that have reported similar windows for optimal sampling [28,29]. While this study provides valuable insights into biomarkers for neurological complications in neonates, it is constrained by several limitations. The sample size, drawn from a single center over one year, and the focus on late preterm and term neonates only may limit the generalizability of the findings. The exclusion of neonates with severe congenital anomalies or genetic syndromes, while necessary, could omit potentially informative cases. Also, data on the oxygenation index and ventilation index were not collected. Additionally, the observational design cannot establish causality between biomarker levels and neurological outcomes, although the prospective design allows us to explore the predictive capabilities of the biomarkers under investigation. Future research could benefit from larger, multicentric studies, including a wider range of neonatal ages and conditions, to enhance the generalizability and robustness of these findings.

## 5. Conclusions

This research establishes TNF-alpha and the NMR as effective early predictors for the development of neurological complications in neonates undergoing mechanical ventilation. The pronounced predictive accuracy of TNF-alpha, particularly when evaluated within the first 72 h post-birth, alongside NMR, emphasizes their critical role in the early identification of neonates at increased risk. The proposed predictive markers are commonly assessed and readily available in clinical settings, and therefore possible to use in every department. By integrating these biomarkers into routine clinical assessments, healthcare providers can significantly enhance their intervention strategies, potentially averting the onset or lessening the severity of neurological complications. This proactive approach underscores a pivotal shift towards precision medicine in neonatal care, where tailored interventions based on individual biomarker profiles can improve overall neurodevelopmental outcomes. Moreover, these findings act as a clarion call for the inclusion of advanced biomarker monitoring in standard neonatal care protocols, advocating for a more nuanced and anticipatory management strategy for neonates in critical care settings. However, to ensure the widespread applicability and reliability of TNF-alpha and NMR as universal biomarkers for neurological risk assessment in mechanically ventilated neonates, it is imperative to conduct extensive multicenter studies.

## Figures and Tables

**Table 1:** Demographics of neonates with and without neurological complications during mechanical ventilation.

Variables	Neurological Complications (*n* = 26)	No Neurological Complications (*n* = 59)	*p*-Value
Gender, *n* (%)			0.895
Male	18 (69.23%)	40 (67.80%)	
Female	8 (30.77%)	19 (32.20%)	
Gestational age, weeks (mean ± SD)	36.69 ± 1.59	36.29 ± 1.82	0.335
Birth weight, grams (mean ± SD)	2941 ± 966	2626 ± 614	0.073
APGAR score 1 min, (mean ± SD)	5 ± 2	7 ± 1	<0.001
APGAR score 5 min, (mean ± SD)	6 ± 1	7 ± 1	<0.001
Monitored pregnancy, *n* (%)	15 (57.69%)	36 (61.02%)	0.773
Cesarean birth, *n* (%)	17 (65.38%)	41 (69.49%)	0.707
Meconium aspiration, *n* (%)	3 (11.54%)	5 (8.47%)	0.655
Transient tachypnea, *n* (%)	8 (30.77%)	23 (38.89%)	0.468

SD—standard deviation; APGAR—appearance, pulse, grimace, activity, and respiration.

**Table 2:** Complications and interventions.

Variables	Neurological Complications (*n* = 26)	No Neurological Complications (*n* = 59)	*p*-Value
Neonatal sepsis, *n* (%)	5 (19.23%)	11 (18.64%)	0.949
Respiratory complications			
ARDS	3 (11.54%)	11 (18.64%)	0.415
Congenital pneumonia	5 (19.23%)	13 (22.03%)	0.770
Neonatal asphyxia	10 (38.46%)	5 (8.47%)	<0.001
Pneumothorax	5 (19.23%)	9 (15.25%)	0.648
Pulmonary hemorrhage	3 (11.54%)	7 (11.86%)	0.965
Recurrent pneumonia	8 (30.77%)	20 (33.90%)	0.777
Neurological complications			
HIE	19 (73.80%)	–	
Intracranial hemorrhage	6 (23.08%)	–	
Seizures	16 (61.54%)	–	
Interventions			
nIPPV	13 (50.00%)	15 (25.42%)	0.026
nCPAP/nIPPV	11 (42.31%)	33 (55.93%)	0.246
SIMV/IPPV	9 (34.62%)	19 (32.20%)	0.827
Days on ventilation (>7)	15 (57.69%)	23 (38.89%)	0.109

ARDS—acute respiratory distress syndrome; HIE—hypoxic ischemic encephalopathy; nIPPV—non-invasive positive pressure ventilation; nCPAP—nasal continuous positive airway pressure; SIMV—synchronized intermittent mechanical ventilation.

**Table 3:** Comparison of baseline (first 24 h) measurements of laboratory data in the neonates with and without neurological complications.

Variables (Mean ± SD) *	Neurological Complications (*n* = 26)	No Neurological Complications (*n* = 59)	*p*-Value **
CRP (0–5 mg/L)	11.39 ± 10.51	8.29 ± 11.58	0.245
Procalcitonin (0–0.5 ng/mL)	16.16 ± 13.27	9.44 ± 15.64	0.059
WBC (9–30 × 10^3^/μL)	20.70 ± 16.79	15.39 ± 5.67	0.032
Lymphocyte count (2–17 × 10^3^/μL)	4.61 ± 2.02	2.41 ± 3.55	0.004
Neutrophil count (1.5–22 × 10^3^/μL)	9.63 ± 6.99	7.20 ± 6.68	0.131
Monocyte count (0.1–1.36 × 10^3^/μL)	2.03 ± 1.65	2.17 ± 4.15	0.868
NMR	7.59 ± 4.09	5.44 ± 4.34	0.035
LMR	6.18 ± 12.06	2.63 ± 1.31	0.027
TNF-alpha (pg/mL)	17.33 ± 15.76	11.06 ± 7.21	0.013

* Normal values were reported based on the hospital’s laboratory normal range; ** Student’s *t*-test; SD—standard deviation; WBC—white blood cell count; CRP—C-reactive protein; NMR—neutrophil-to-monocyte ratio; LMR—leukocyte-to-monocyte ratio; TNF—tumor necrosis factor.

**Table 4:** Comparison of 72 h measurements of laboratory data in the neonates with and without neurological complications.

Variables (Mean ± SD) *	Neurological Complications (*n* = 26)	No Neurological Complications (*n* = 59)	*p*-Value **
CRP (0–5 mg/L)	12.64 ± 12.91	6.89 ± 10.85	0.036
Procalcitonin (0–0.5 ng/mL)	8.56 ± 7.44	4.43 ± 8.94	0.042
WBC (9–30 × 10^3^/μL)	17.35 ± 10.47	13.51 ± 4.52	0.020
Lymphocyte count (2–17 × 10^3^/μL)	4.59 ± 2.06	3.66 ± 3.24	0.181
Neutrophil count (1.5–22 × 10^3^/μL)	7.58 ± 4.53	3.41 ± 2.91	<0.001
Monocyte count (0.1–1.36 × 10^3^/μL)	2.50 ± 3.04	1.87 ± 0.88	0.145
NMR	5.42 ± 3.47	3.83 ± 3.35	0.049
LMR	4.22 ± 6.09	2.71 ± 2.00	0.005
TNF-alpha (pg/mL)	19.59 ± 9.57	13.65 ± 14.35	0.022

* Normal values were reported based on the hospital’s laboratory normal range; ** Student’s *t*-test; SD—standard deviation; WBC—white blood cell count; CRP—C-reactive protein; NMR—neutrophil-to-monocyte ratio; LMR—leukocyte-to-monocyte ratio; TNF—tumor necrosis factor.

**Table 5:** Best cutoff values for predicting neurological complications.

Laboratory Parameter	Timeframe	Best Cutoff Value	Sensitivity	Specificity	AUC	*p*-Value
CRP	24 h	>10.7 mg/L	68%	62%	0.431	0.168
Procalcitonin	24 h	>12.2 ng/mL	66%	59%	0.429	0.183
NMR	24 h	>5.3	78%	67%	0.562	0.029
LMR	24 h	>4.2	71%	64%	0.558	0.038
TNF-alpha	24 h	>12.8 pg/mL	82%	69%	0.574	0.005
CRP	72 h	>15.4 mg/L	74%	66%	0.570	0.032
Procalcitonin	72 h	>0.32 ng/mL	70%	61%	0.520	0.059
NMR	72 h	>6.1	76%	68%	0.567	0.025
LMR	72 h	>3.7	68%	63%	0.510	0.076
TNF-alpha	72 h	>14.3 pg/mL	87%	72%	0.593	<0.001

CRP—C-reactive protein; NMR—neutrophil-to-monocyte ratio; LMR—leukocyte-to-monocyte ratio; TNF—tumor necrosis factor; AUC—area under the curve.

**Table 6:** Regression analysis for neurological complications development.

Factors above the Best Cutoff	Hazard Ratio	95% CI	*p*-Value
CRP	1.41	1.06–4.81	0.030
Procalcitonin	1.30	0.94–3.17	0.093
NMR	2.16	1.18–4.09	0.022
LMR	1.94	1.32–4.26	0.008
TNF-alpha	3.32	2.06–6.39	<0.001

CRP—C-reactive protein; NMR—neutrophil-to-monocyte ratio; LMR—leukocyte-to-monocyte ratio; TNF—tumor necrosis factor; CI—confidence interval.

## Data Availability

Data available on request from the authors.
